# Downregulation of HADH promotes gastric cancer progression via Akt signaling pathway

**DOI:** 10.18632/oncotarget.19348

**Published:** 2017-07-18

**Authors:** Congcong Shen, Yao-Hua Song, Yufeng Xie, Xiaoxiao Wang, Yunliang Wang, Chao Wang, Songbai Liu, Sheng-Li Xue, Yangxin Li, Bin Liu, Zaixiang Tang, Weichang Chen, Jenny Song, Hesham M. Amin, Jin Zhou

**Affiliations:** ^1^ Cyrus Tang Hematology Center, Collaborative Innovation Center of Hematology, Soochow University, Suzhou, P. R. China; ^2^ Department of Oncology, The First Affiliated Hospital of Soochow University, Suzhou, P. R. China; ^3^ Department of General Surgery, The First Affiliated Hospital of Soochow University, Suzhou, P. R. China; ^4^ Department of Gastroenterology, The First Affiliated Hospital of Soochow University, Suzhou, P. R. China; ^5^ Suzhou Vocational Health College, Suzhou Key Laboratory of Biotechnology for Laboratory Medicine, Suzhou, P. R. China; ^6^ Department of Hematology, The First Affiliated Hospital of Soochow University, Jiangsu Institute of Hematology, Collaborative Innovation Center of Hematology, Soochow University, Suzhou, P. R. China; ^7^ Department of Cardiovascular Surgery & Institute of Cardiovascular Science, First Affiliated Hospital of Soochow University, Suzhou, P. R. China; ^8^ Department of Cardiology, Second Hospital of Jilin University, Changchun, P. R. China; ^9^ Department of Biostatistics, School of Public Health, Medical College of Soochow University, Suzhou, P. R. China; ^10^ Department of Hematopathology, The University of Texas MD Anderson Cancer Center, Houston, Texas, USA

**Keywords:** gastric cancer, HADH, fatty acids, migration, invasion

## Abstract

HADH is a key enzyme in fatty acid oxidation. The aim of this study was to identify the role of HADH in gastric cancer. We analyzed the expression of HADH in 102 pairs of gastric cancer samples. Western blot analysis revealed that HADH was decreased in stage I/II gastric cancer samples compared to matched adjacent normal gastric tissue, and its expression was further decreased in stage III/IV samples. Importantly, the reduced expression of HADH was associated with increased expression of p-Akt and reduced expression of PTEN in the gastric carcinoma tumor samples. To determine the significance of HADH downregulation in gastric cancer progression, we tested the impact of HADH knockdown or overexpression on the migration and invasion of the gastric cancer cells using a transwell assay. Knockdown of HADH significantly promoted gastric cancer cell migration and invasion, which was associated with increased expression of p-Akt. The PI3K inhibitor LY294002 inhibited HADH shRNA induced migration/invasion, and abolished the upregulation of p-Akt. By contrast, HADH overexpression inhibited the migration and invasion of MKN45 cells. Herein, for the first time, we demonstrate that downregulation of HADH promotes gastric cancer progression via activation of Akt signaling pathway.

## INTRODUCTION

Proliferating cancer cells require lipid for cellular membrane synthesis and other essential functions [1]. Numerous studies have shown that the expression of enzymes involved in fatty acid synthesis is increased in gastric cancer (GC)[2–6]. However, the role of fatty acid breakdown in cancer cells remains controversial. Fatty acid breakdown is carried out in the mitochondria through β-oxidation, which is a multistep process involving 4 major enzymes: acyl-CoA dehydrogenase, enoyl-CoA hydratase, hydroxyacyl-CoA dehydrogenase, and ketoacyl-CoA thiolase. It was shown that enoyl coenzyme A hydratase short chain 1, an important enzyme in the second step of β-oxidation, was highly expressed in both gastric cancer cell lines [7] and in tissue samples of GC [8]. However, a recent study showed that long-chain acyl-CoA dehydrogenases (LCAD), one of the enzymes involved in the first oxidation step, actually suppresses the growth of hepatocellular carcinoma [9].

It was shown that decreased LCAD expression predicts patient mortality [9]. The expression of hydroxyacyl-CoA dehydrogenase was significantly under-expressed in breast cancer; especially in those with estrogen receptor-negative status and those with metastatic and recurring breast cancers [10]. Differential carbonylation analysis showed protein damage in 3-ketoacyl-CoA thiolase in human hepatocellular carcinoma [11].

Clinical studies showed that the mRNA levels of several enzymes involved in β-oxidation were decreased in cancer [12, 13]. These findings prompted us to investigate the expression of 3-hydroxyacyl-CoA dehydrogenase (HADH) in GC. HADH is an enzyme located in the mitochondria, and is essential to convert short and medium chain fatty acids into ketones to provide energy for liver, heart, muscle, and pancreas during prolonged period of fasting. The enzyme is encoded by *HADH* gene and mutations of this gene cause hyperinsulinemic hypoketotic hypoglycemia [14]. However, the role of HADH in gastric cancer progression remains unknown. In the present study, we analyzed the expression of HADH by Western blot and immunofluorecence staining. We also examined the role of HADH in gastric cancer cell migration and invasion using a transwell system and identified the signaling pathways mediating HADH's effect.

## RESULTS

### HADH protein expression is decreased in human GC

**Figure 1:**
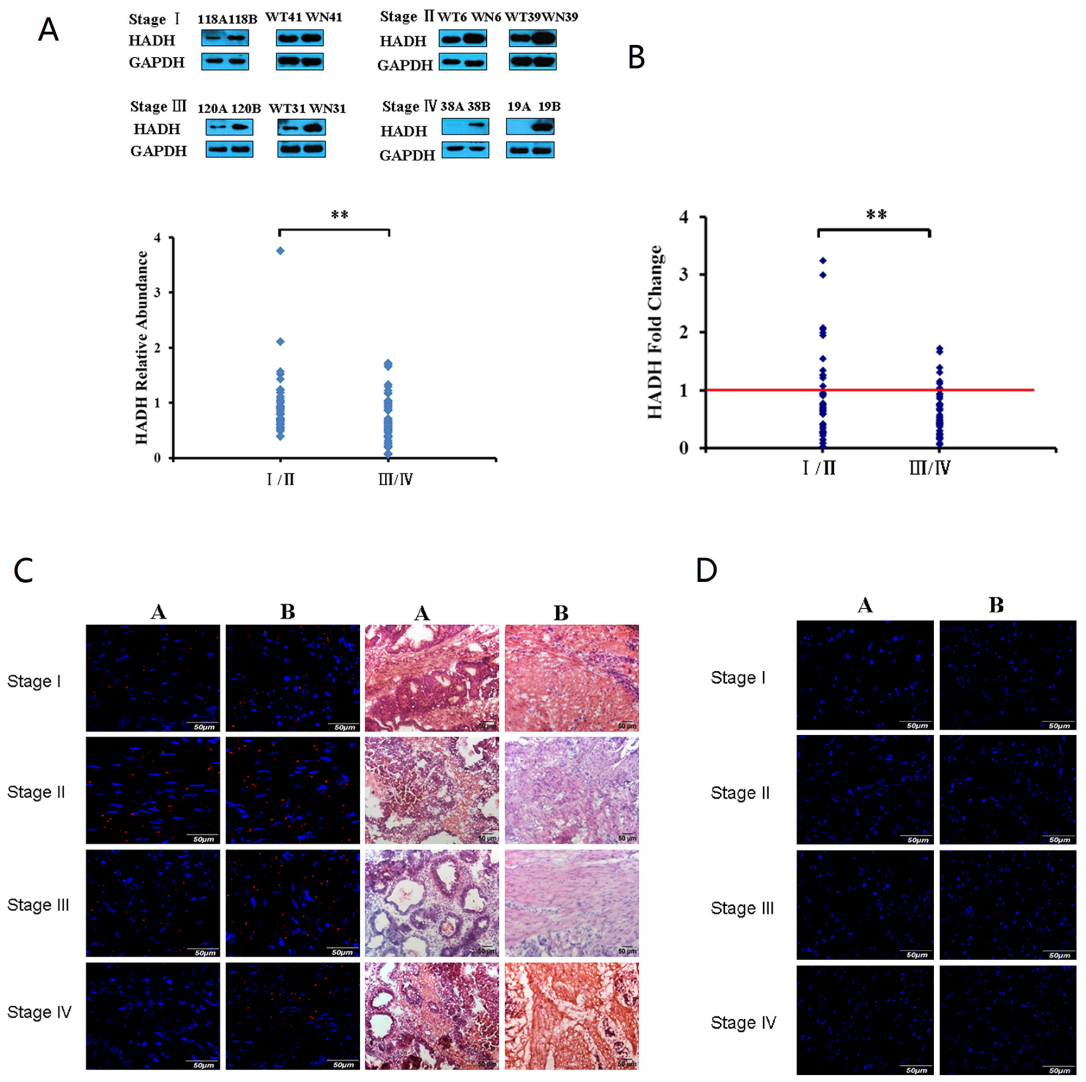
HADH expression was analyzed in GC samples and matched adjacent normal gastric tissues by Western blot, real-time PCR, and immunofluorescence techniques **(A)** Top panel: representative Western blot showing HADH expression in stages I-IV GC samples and their matched adjacent normal gastric tissues. 118A, 120A, 38A, 19A, WT41, WT31, WT6 and WT39 are clinical samples from patients with gastric cancer. 118B, 120B, 38B, 19B, WN41, WN31, WN6 and WN39 are matched adjacent normal gastric tissue. Lower panel: HADH relative abundance in 102 GC samples. The bands were quantified by ImageJ and normalized to GAPDH. The normalized densities from GC samples were then divided by that of the corresponding adjacent normal gastric tissues. **P < 0.01. **(B)** Real-time PCR analysis of HADH mRNA levels. HADH mRNA levels from 102 pairs of GC samples were detected by real-time PCR. **(C)** Validation of HADH expression by immunofluorescence staining. Frozen sections from stage I-IV GC (A or WT) and their adjacent normal gastric tissues (B or WN) were incubated with an antibody against human HADH, followed by goat anti-rabbit IgG - Alexa Fluor® 568 conjugate. Nuclei are stained with DAPI (blue), HE staining sections from the same tissue are also provided to identify the location of the sections. **(D)** Negative control, the frozen sections were incubated with normal rabbit IgG, followed by goat anti-rabbit IgG - Alexa Fluor® 568 conjugate.

We studied HADH expression in 102 human tissue samples that included GC and adjacent normal gastric tissues. The patients comprised 73 males and 29 females covering all four stages of GC (Table 1). Western blot assay demonstrated that HADH expression was abundant in normal gastric tissue, weak in stage I-II GC, and barely detectable in stage III-IV GC tumors (Figure 1A), suggesting that HADH expression gradually decreases as GC progresses to a more advanced clinical stage. Real-time PCR results revealed a similar change at the transcription level (Figure 1B). Next, we used immunofluorescence staining to examine the expression of HADH in frozen tissue sections from the above mentioned samples. Consistently, immunofluorescence staining also showed that HADH expression was reduced in GC samples compared with the adjacent normal gastric tissue (Figure 1C-1D). The Western blot data for all 102 pairs of GC samples are shown in Supplementary Figure 1.

**Table 1:** Demographic characteristics of GC patients for Western blot analysis

	Age		Gender		Histological grade		Stage			
	>64	≤64	Male	Female	Moderate differentiation	Poor differentiation	I	II	III	IV
N(%)	56(54.9)	46(45.1)	73(71.6)	29(28.4)	44(43.1)	58(56.9)	19(18.6)	28(27.5)	43(42.2)	12(11.8)

### HADH knockdown promotes migration and invasion of GC cells

**Figure 2:**
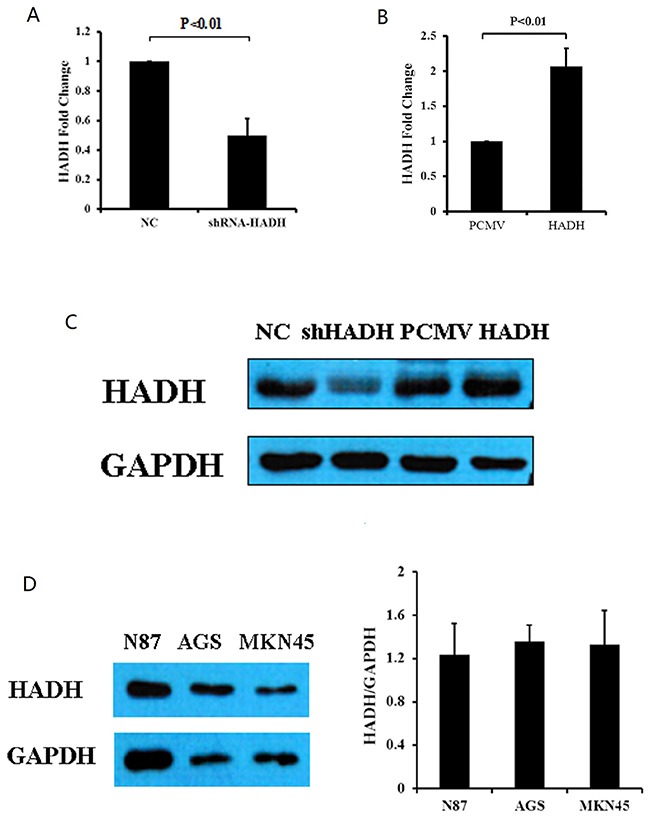
HADH expression in gastric cancer lines **(A)** HADH mRNA levels were reduced in MKN45 cells transfected with HADH shRNA compared to NC. N = 4. **(B)** HADH mRNA level increased in MKN45 cells transfected with an HADH expression plasmid compared to cells transfected with vector alone. N = 3. **(C)** Western blot analysis of HADH expression in MKN45 cells transfected with HADH shRNA (shHADH), control non targeting lentivirus (NC), HADH over expression plasmid (HADH), or control plasmid (PCMV). **(D)** Western blot analysis of HADH expression in three gastric cancer lines. Basal HADH level was detected in N87, AGS and MKN45 cells. N = 3.

We next investigated the impact of HADH knockdown or overexpression on GC cell migration and invasion. HADH knockdown or overexpression was achieved by transfecting MKN45 gastric cancer cells with shRNA or plasmid carrying full length human HADH cDNA, respectively. Real-time PCR results demonstrated that HADH mRNA expression was significantly reduced in the MKN45 cells transfected with shRNA (Figure 2A), whereas its expression was increased in the cells that were transfected with an expression plasmid carrying HADH cDNA (Figure 2B). The mean of shRNA-HADH and HADH is 0.501±+0.11 and 2.06±0.25, respectively. Transfection efficiency was also confirmed by Western blot analysis (Figure 2C). We analyzed HADH basal expression level in N87, AGS and MKN45 gastric cancer lines and our results showed similar moderate HADH expression in all three cell lines (Figure 2D).

**Figure 3:**
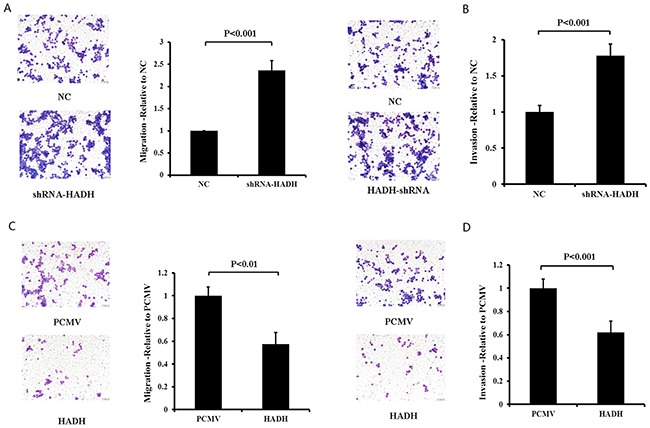
The effect of HADH knockdown and overexpression on MKN45 migration and invasion **(A)** Migration assay was conducted in HADH shRNA- or NC-transfected MKN45 cells by using 24-well Transwell chambers. Representative images of migrated cells after transfection of NC or shRNA. Cell migration was assessed by counting the number of MKN45 cells that migrated through the transwell insert in 3 independent membranes by using light microscopy, and then normalized against the NC-treated cells to determine the relative ratio. **(B)** Knockdown of HADH by shRNA promotes MKN45 cell invasion. Invasion assay was conducted in HADH shRNA- or NC-transfected MKN45 cells using 24-well Transwell chambers. The procedures for cell invasion are similar to that of migration except that matrigel was added to the upper chamber of the transwell. **(C)** Overexpression of HADH in MKN45 cells inhibits migration. Migration assay was performed in MKN45 cells transfected with an expression plasmid carrying HADH cDNA or the empty vector PCMV using 24-well Transwell chambers. **(D)** Overexpression of HADH in MKN45 cell inhibits invasion. Invasion assay was conducted in MKN45 cells transfected with an expression plasmid carrying HADH cDNA or the empty vector PCMV using 24-well Transwell chambers.

We then performed migration and invasion assays using transwell plates. Our data revealed that HADH knockdown enhanced the migration and invasion capacity of MKN45 cells compared with control cells (Figure 3A–3B). By contrast, HADH overexpression inhibited the migration and invasion of these cells (Figure 3C–3D).We thenperformed these experiments using two additional gastric cancer lines AGS and N87 cells and the data confirmed that HADH knockdown promotes gastric cancer cell invasion and migration (Supplementary Figure 2).

**Figure 4:**
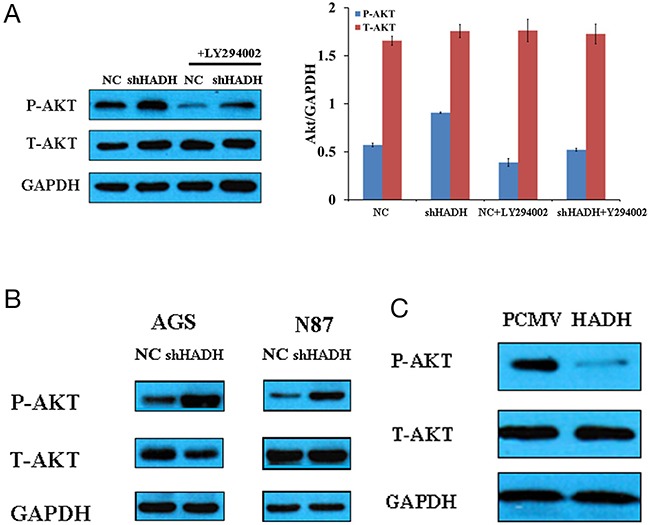
Western blot assay of Akt expression **(A)** Representative Western blots of p-Akt, t-Akt, and GAPDH expression in lysates of MKN45 cells transfected with HADH shRNA in the presence or absence of 20 μM LY294002. **(B)** Representative Western blots of p-Akt, t-Akt, and GAPDH expression in lysates of N87 and AGS cells transfected with HADH shRNA or non targeting lentivirus (NC). **(C)** Representative Western blots of p-Akt, t-Akt, and GAPDH expression in lysates of MKN45 cells transfected with plasmid overexpressing HADH or control vector (PCMV).

Because Akt signaling pathway is involved in tumor progression and invasion [15–18], we investigated whether there is a causal relationship between HADH downregulation and increased p-Akt upregulation in MKN45 cells. Our data show that p-Akt level was increased, while total Akt level was unchanged, in MKN45, N87, and AGS cells transfected with HADH shRNA (Figure 4A–4B). By contrast, p-Akt level was decreased in MKN45 cells transfected with plasmid overexpressing HADH (Figure 4C). Furthermore, HADH shRNA-induced p-Akt upregulation was hindered in the presence of the PI3K inhibitor LY294002 (Figure 4A). These results suggest that the downregulation of HADH led to activation of Akt signaling pathway in MKN45 cells.

**Figure 5:**
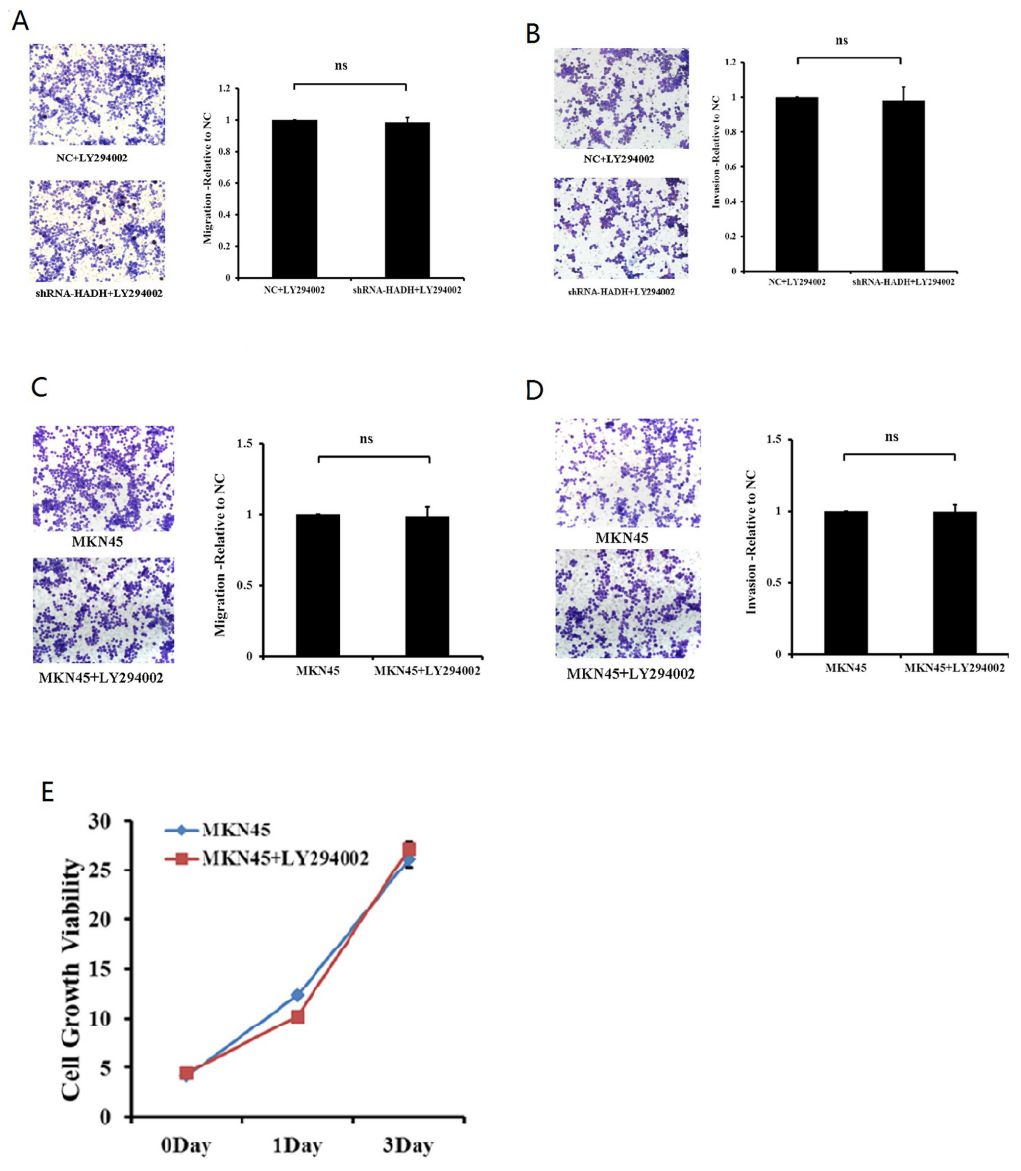
The PI3K inhibitor prevents HADH knockdown-induced MKN45 cell migration **(A)** Migration assay was performed with HADH shRNA- or NC-transfected MKN45 cells in the presence of 20 μM LY294002 using 24-well Transwell chambers. The cells were treated with LY294002 for 24 hours at a final concentration of 20 μM prior to transfection. Cell migration was assessed by counting the number of MKN45 cells that migrated through the transwell insert in 3 independent membranes by using light microscopy, and then normalized against the NC-treated cells to determine the relative ratio. ns: not significant. **(B)** Invasion assay was performed with HADH shRNA- or NC-transfected MKN45 cells in the presence of 20 μM LY294002 using 24-well Transwell chambers. **(C-D)** LY294002 has no effect on migration and invasion for MKN45 cells that were not transfected with HADH shRNA. MKN45 migration and invasion were performed in the presence or absence of 20 μM LY294002. **(E)** LY294002 has no effect on proliferation for MKN45 cells that were not transfected with HADH shRNA. MKN45 proliferation was performed in the presence or absence of 20 μM LY294002 was assessed by CCK8 reagent.

To determine the role of Akt activation in tumor migration and invasion, we added the PI3K inhibitor LY294002 to the culture media of MKN45 cells transfected with HADH shRNA in the transwell. LY294002 inhibited HADH shRNA-induced migration and invasion (Figure 5A–5B). However, LY294002 has no effect on migration, invasion and proliferation for MKN45 cells that were not transfected with HADH shRNA (Figure 5C–5E).

**Figure 6:**
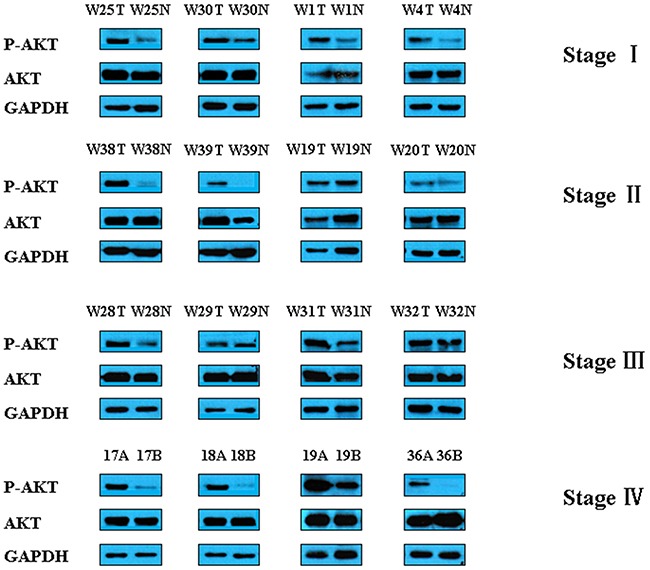
Representative Western blot images of p-Akt and t-Akt in gastric cancer tissues The expression of Akt was analyzed in GC samples and matched adjacent normal gastric tissues by Western blot.

We then analyzed the expression of Akt in GC tumors and adjacent normal tissues from patients. As shown in Figure 6, p-Akt level is increased in GC samples compared with matched controls, but total Akt levels were similar between GC and control tissues. Taken together, these data suggest that the downregulation of HADH promotes gastric cancer progression through the activation of Akt signaling pathway.Because Akt is negatively regulated by PTEN, the expression of PTEN was detected by qPCR and Western blot (Supplementary Figure 3). The results showed that PTEN expression is reduced in gastric cancer samples compared to adjacent normal gastric tissues.

### HADH knockdown promotes proliferation of gastric cancer cells

**Figure 7:**
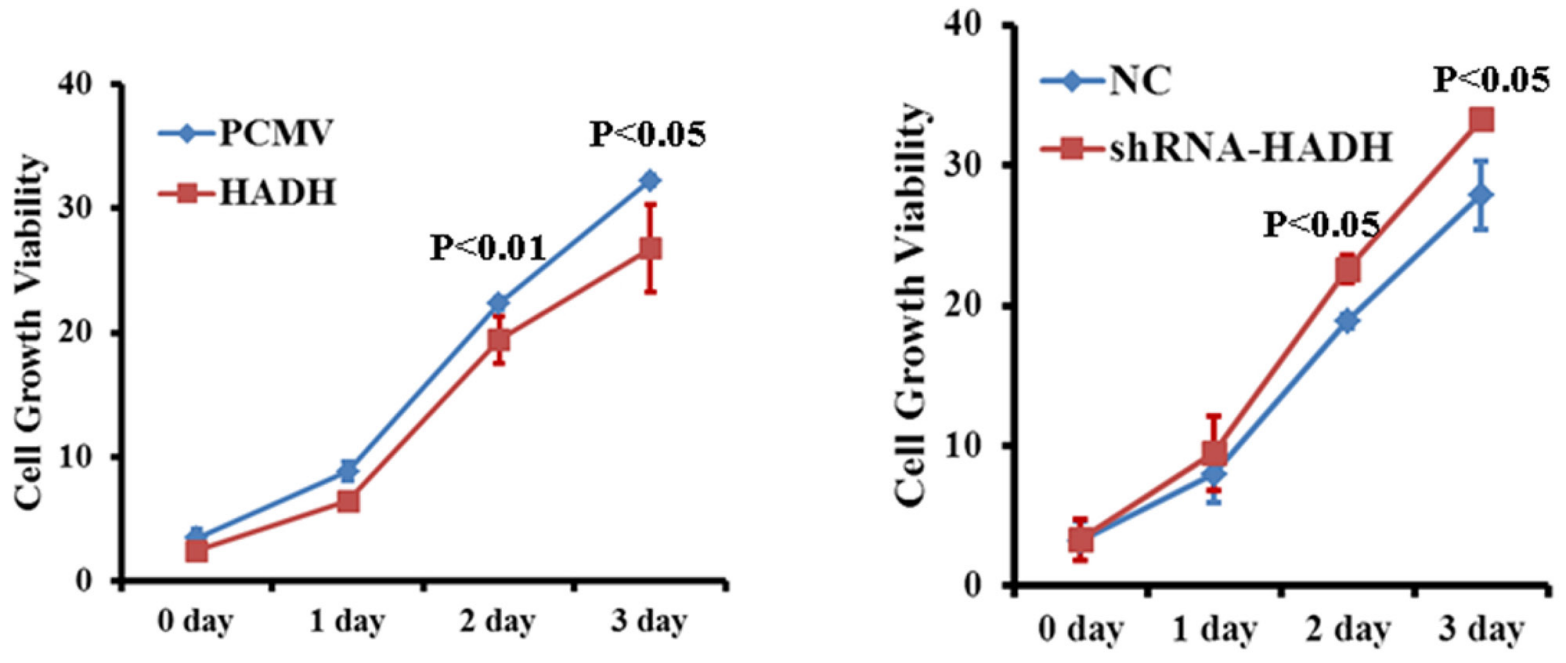
Effect of HADH knockdown on gastric cancer cell proliferation Cell proliferation was assessed in MKN45 cells transfected with either HADH shRNA lentivirus or HADH over expression plasmid. MKN45 cells transfected with NC and PCMV vectors were used as control.

Because we have shown HADH down regulation correlates tumor progression in most of the GC samples, we then investigated whether HADH knockdown or overexpression affects gastric cancer cell proliferation *in vitro*. Cell proliferation assay revealed that HADH shRNA promoted MKN45 cell growth compared to nontargeting control shRNA (Figure 7). By contrast, HADH over expression inhibit growth of gastric cancer cells compared to cells transfected with empty plasmid (Figure 7). Therefore, some of the cells that we observed under migration and invasion experiment might be due to increased proliferation.

## DISCUSSION

In the present study, we analyzed protein expression of HADH in 102 GC samples and found that the expression of this enzyme was decreased in stage I/II GC samples compared to matched adjacent normal gastric tissue, and its expression further decreased in stage III/IV samples. We showed that HADH downregulation was associated with increased expression of p-Akt and reduced expression of PTEN in GC samples. Knockdown of HADH promoted the proliferation, migration and invasion of the gastric cancer cells MKN45 via activation of Akt signaling pathway.

How does HADH influence Akt signaling? The downregulation of HADH could slow down β-oxidation, which leads to accumulation of fatty acids that inhibit the transcription of PTEN [9]. PTEN is a major negative regulator of the PI3K/Akt signaling pathway. Therefore, PTEN repression will lead to Akt activation in cells with HADH knockdown. Activation of Akt signaling pathway promotes proliferation and invasion of gastric cancer cells [19].

The accumulation of fatty acids could also result from increased activity of lipogenic enzymes such as fatty acid synthase (FASN). Elevated expression of FASN is associated with advanced stage colorectal cancer and colon cancer metastasis [20]. FASN-induced tumor progression is mediated by CD44, a transmembrane protein that promotes tumor invasion via activation of Akt signaling pathway [20, 21]. Conversely, inhibition of FASN suppresses invasion and migration via downregulation of the activity of Her2/PI3K/Akt signaling in osteosarcoma cells [22, 23].

In conclusion, our study demonstrates that the expression of HADH is decreased during gastric cancer progression. Moreover, our data show that HADH knockdown promotes tumor cell migration and invasion through activation of Akt signaling pathway.

## MATERIALS AND METHODS

### Patient samples

All experiments involving human subjects were performed in accordance with the Code of Ethics of the World Medical Association (Declaration of Helsinki), and the relevant guidelines and regulations of Soochow University. All experimental protocols were approved by the Research Ethics Committee of the First Affiliated Hospital of Soochow University. With informed consents from all subjects, paired specimens of GC and adjacent normal tissues were obtained from patients who underwent surgical resection of GC at the Department of General Surgery, First Affiliated Hospital of Soochow University. None of the patients received any anti-cancer treatment before surgery.

### Western blot

Proteins extracted from patients’ tumor tissue samples were separated by SDS-PAGE and then transferred to PVDF membrane. After incubation with primary antibodies at 4°C overnight, the membranes were washed three times with Tris-buffered saline containing Tween-20 (TBST), and then incubated with the horseradish peroxidase-conjugated secondary antibody (anti-rabbit IgG: 1:4000, Sigma, USA) for 2 h at room temperature. The membranes were washed again in TBST and visualized using an Enhanced Chemiluminescence Kit (PerkinElmer). The rabbit anti-HADH (1/1000) was from Thermo Fisher Scientific. The anti-p-Akt, Akt (Ser-473, 1/1000), and GAPDH antibodies (1/8000) were from Cell Signaling. The band density was quantified by ImageJ and normalized to GAPDH.

### Immunofluorescence staining

Frozen sections (10 μm) were incubated with HADH antibody produced in rabbit (1/500; ThermoFisher Scientific). After washing, the sections were incubated with goat anti-rabbit IgG - Alexa Fluor® 568 conjugate (Thermo Fisher Scientific), followed by incubation with DAPI to stain nuclei. Images were acquired using a Multiphoton Laser Scanning Microscope (FV1000, Olympus).

### Cell lines

The human gastric carcinoma cell line MKN45 was purchased from China Infrastructure of Cell Line Resources (Beijing, China), and cultured in RPMI containing 20% FBS. The human gastric carcinoma cell lines AGS and N87 were purchased from Cell Bank of the Chinese Academy of Sciences (Shanghai, China). AGS cells were cultured in Ham's F-12K (Gibco) containing 10% FBS. The N87 cells were cultured in RPMI 1640 supplemented with 10% FBS.

### Cell proliferation assay

The plasmids carrying HADH shRNA or HADH cDNA (3 μg) were diluted in 150 μl Opti-MEM which were then mixed with equal volumes of Opti-MEM containing 6 μl Lipofectamine 2000. The mixture was incubated for 20 minutes at room temperature before adding to MKN45 cells (4.5 × 10^5^ cells/well) in a 6-well plate. After 24 hours incubation, the cells were digested with trypsin and then re-seeded to 96-well plates (5000 cells/well) and 10μl CCK8 reagent (Beyotime Biotech, Shanghai) was added to each well. Absorbance was detected at 0h,24h,48h,72h at 450 nm using a Multi-Detection Reader.

### HADH overexpression

The expression plasmid (pCMV3) carrying HADH was purchased from Sino Biological Inc (Beijing, China). Transient transfection was mediated by Lipofectamine 2000. The plasmid (3 μg) was diluted in 150 μl Opti-MEM which were then mixed with equal volumes of Opti-MEM containing 6 μl Lipofectamine 2000. The mixture was incubated for 20 minutes at room temperature before adding it to the cells. For migration and invasion experiments involving LY294002, the cells were treated with LY294002 for 24 hours at a final concentration of 20 μM prior to transfection.

### Real-time PCR

RNA was extracted using the Trizol reagent (Ambion), and cDNA was synthesized using the RevertAid First Strand cDNA Synthesis Kit (ThermoFisher Scientific). Real-time PCR was performed using the ABI 7500 Real-time PCR System with the following primers:

HADH-F: GCTTCTAGATTATGTCGGACTGG,

HADH-R: TGGGCTGATGTAATGGGTTC,

GAPDH-F: ACCCAGAAGACTGTGGATGG,

GAPDH-R: CAGTGAGCTTCCCGTTCAG.

Relative expression was calculated from cycle threshold (Ct; relative expression = 2^–(SΔCt – CΔCt)^) values using GAPDH as internal control for each samples.

### HADH shRNA transfection

For knockdown of HADH, MKN45 cells were transfected with shRNA targeting HADH. shRNA and negative control (NC) were purchased from GenePharma (Shanghai, China). HADH shRNA: GGACTGGATAC TACGAAGTTC, NC: TTCTCCGAACGTGTCACGT

The cells were transfected according to the manufacturer's instructions. MKN45 cells were seeded to 6-well plates (4.5 × 10^5^ cells/well) the night before transfection. The transfection was designed for one RNA amount (A) combined with one amount of Lipofectamine 2000 (B). A. 3 μg shRNA plasmid was added to 150 μl Opti-MEM; B. 6 μl Lipofectamine 2000 (Invitrogen), was added to 150 μl Opti-MEM. A and B was combined and incubated at room temperature for 20 mins. Finally, 250 μl of the mixture was added to each well of the 6-well plates.

### Migration and invasion assays

Cell invasion was determined using a transwell matrigel invasion assay in 24-well Transwell units (Costar)[24]. Matrigel diluted with the precooled serum-free RPMI (50 μl) was added to the upper chamber of the Transwell and incubated at 37°C for 30 minutes. The shRNAs or NC was prepared as described above. After 24 hours incubation, the cells were digested with trypsin, resuspended in 200 μl serum-free RPMI and transferred to Matrigel coated top chambers (1 × 10^5^ cells/well). The lower chambers were filled with 500 μl RPMI supplemented with 10% FBS. After incubation at 37°C for 24 hours, the non-invading cells were removed with a cotton swab. The inserts were removed from the top chambers, washed with PBS, fixed and stained with Giemsa. The invaded cells were counted in five random fields under a light microscope. The procedure for cell migration is similar to that of invasion, except that matrigel was not added.

### Statistical analysis

Data were presented as means ± SD. The t*-*test was used to determine the significance of the differences between two groups for the migration and invasion assay. Mann-whitney test was used to determine the significance for Westernblot experiments. *P* < 0.05 was considered statistically significant.

## SUPPLEMENTARY MATERIALS FIGURES



## Abbreviations

HADH: 3-hydroxyacyl-CoA dehydrogenase
ECHS1: enoyl coenzyme A hydratase short chain 1
GC: gastric carcinoma
LCAD: long-chain acyl-CoA dehydrogenases
FASN: fatty acid synthase

## References

[1] Martinez-Outschoorn UE, Peiris-Pages M, Pestell RG, Sotgia F, Lisanti MP (2017). Cancer metabolism: a therapeutic perspective. Nat Rev Clin Oncol.

[2] Fang W, Cui H, Yu D, Chen Y, Wang J, Yu G (2014). Increased expression of phospho-acetyl-CoA carboxylase protein is an independent prognostic factor for human gastric cancer without lymph node metastasis. Med Oncol.

[3] Qian X, Hu J, Zhao J, Chen H (2015). ATP citrate lyase expression is associated with advanced stage and prognosis in gastric adenocarcinoma. Int J Clin Exp Med.

[4] Duan J, Sun L, Huang H, Wu Z, Wang L, Liao W (2016). Overexpression of fatty acid synthase predicts a poor prognosis for human gastric cancer. Mol Med Rep.

[5] Ito T, Sato K, Maekawa H, Sakurada M, Orita H, Shimada K, Daida H, Wada R, Abe M, Hino O, Kajiyama Y (2014). Elevated levels of serum fatty acid synthase in patients with gastric carcinoma. Oncol Lett.

[6] Li HE, Wang X, Tang Z, Liu F, Chen W, Fang Y, Wang C, Shen K, Qin J, Shen Z, Sun Y, Qin X (2015). A concordant expression pattern of fatty acid synthase and membranous human epidermal growth factor receptor 2 exists in gastric cancer and is associated with a poor prognosis in gastric adenocarcinoma patients. Oncol Lett.

[7] Zhu XS, Gao P, Dai YC, Xie JP, Zeng W, Lian QN (2014). Attenuation of enoyl coenzyme A hydratase short chain 1 expression in gastric cancer cells inhibits cell proliferation and migration *in vitro*. Cell Mol Biol Lett.

[8] Zhang J, Song M, Wang J, Sun M, Wang B, Li R, Huang Y, Hou L, Jin Y, Wang M, Tang J (2011). Enoyl coenzyme A hydratase 1 is an important factor in the lymphatic metastasis of tumors. Biomed Pharmacother.

[9] Huang D, Li T, Li X, Zhang L, Sun L, He X, Zhong X, Jia D, Song L, Semenza GL, Gao P, Zhang H (2014). HIF-1-mediated suppression of acyl-CoA dehydrogenases and fatty acid oxidation is critical for cancer progression. Cell Rep.

[10] Mamtani M, Kulkarni H (2012). Association of HADHA expression with the risk of breast cancer: targeted subset analysis and meta-analysis of microarray data. BMC Res Notes.

[11] Martin FA, Mebarki M, Paradis V, Friguet B, Radman M (2014). Hepatocellular carcinoma protein carbonylation in virus C and metabolic syndrome patients. Free Radic Biol Med.

[12] Tanaka M, Masaki Y, Tanaka K, Miyazaki M, Kato M, Sugimoto R, Nakamura K, Aishima S, Shirabe K, Nakamuta M, Enjoji M, Kotoh K, Takayanagi R (2013). Reduction of fatty acid oxidation and responses to hypoxia correlate with the progression of de-differentiation in HCC. Mol Med Rep.

[13] Enjoji M, Kohjima M, Ohtsu K, Matsunaga K, Murata Y, Nakamuta M, Imamura K, Tanabe H, Iwashita A, Nagahama T, Yao K (2016). Intracellular mechanisms underlying lipid accumulation (white opaque substance) in gastric epithelial neoplasms: a pilot study of expression profiles of lipid-metabolism-associated genes. J Gastroenterol Hepatol.

[14] Popa FI, Perlini S, Teofoli F, Degani D, Funghini S, La Marca G, Rinaldo P, Vincenzi M, Antoniazzi F, Boner A, Camilot M (2012). 3-hydroxyacyl-coenzyme a dehydrogenase deficiency: identification of a new mutation causing hyperinsulinemic hypoketotic hypoglycemia, altered organic acids and acylcarnitines concentrations. JIMD Rep.

[15] Xiao J, Sun Q, Bei Y, Zhang L, Dimitrova-Shumkovska J, Lv D, Yang Y, Cao Y, Zhao Y, Song M, Song Y, Wang F, Yang C (2016). Therapeutic inhibition of phospholipase D1 suppresses hepatocellular carcinoma. Clin Sci (Lond).

[16] He X, Liu Z, Xia Y, Xu J, Lv G, Wang L, Ma T, Jiang L, Mou Y, Jiang X, Ma J, Zhao Z, Ni H (2017). HOXB7 overexpression promotes cell proliferation and correlates with poor prognosis in gastric cancer patients by inducing expression of both AKT and MARKs. Oncotarget.

[17] Zhou H, Wu J, Wang T, Zhang X, Liu D (2016). CXCL10/CXCR3 axis promotes the invasion of gastric cancer via PI3K/AKT pathway-dependent MMPs production. Biomed Pharmacother.

[18] Zang M, Zhang B, Zhang Y, Li J, Su L, Zhu Z, Gu Q, Liu B, Yan M (2014). CEACAM6 promotes gastric cancer invasion and metastasis by inducing epithelial-mesenchymal transition via PI3K/AKT signaling pathway. PLoS One.

[19] Yin K, Wang L, Zhang X, He Z, Xia Y, Xu J, Wei S, Li B, Li Z, Sun G, Li Q, Xu H, Xu Z (2017). Netrin-1 promotes gastric cancer cell proliferation and invasion via the receptor neogenin through PI3K/AKT signaling pathway. Oncotarget.

[20] Zaytseva YY, Rychahou PG, Gulhati P, Elliott VA, Mustain WC, O'Connor K, Morris AJ, Sunkara M, Weiss HL, Lee EY, Evers BM (2012). Inhibition of fatty acid synthase attenuates CD44-associated signaling and reduces metastasis in colorectal cancer. Cancer Res.

[21] Yu S, Cai X, Wu C, Wu L, Wang Y, Liu Y, Yu Z, Qin S, Ma F, Thiery JP, Chen L (2015). Adhesion glycoprotein CD44 functions as an upstream regulator of a network connecting ERK, AKT and Hippo-YAP pathways in cancer progression. Oncotarget.

[22] Wang TF, Wang H, Peng AF, Luo QF, Liu ZL, Zhou RP, Gao S, Zhou Y, Chen WZ (2013). Inhibition of fatty acid synthase suppresses U-2 OS cell invasion and migration via downregulating the activity of HER2/PI3K/AKT signaling pathway *in vitro*. Biochem Biophys Res Commun.

[23] Liu ZL, Mao JH, Peng AF, Yin QS, Zhou Y, Long XH, Huang SH (2013). Inhibition of fatty acid synthase suppresses osteosarcoma cell invasion and migration via downregulation of the PI3K/Akt signaling pathway *in vitro*. Mol Med Rep.

[24] Tang N, Shi L, Yu Z, Dong P, Wang C, Huo X, Zhang B, Huang S, Deng S, Liu K, Ma T, Wang X, Wu L, Ma XC (2016). Gamabufotalin, a major derivative of bufadienolide, inhibits VEGF-induced angiogenesis by suppressing VEGFR-2 signaling pathway. Oncotarget.

